# A Plea for Keener Observation, More Conservatism and a Better Understanding of the Question, “Which Is the Best Alloy?”

**Published:** 1908-08-15

**Authors:** Marcus L. Ward


### A PLEA FOR KEENER OBSERVATION, MORE CONSERVATISM AND A BETTER UNDERSTANDING OF THE QUESTION, "WHICH IS THE BEST ALLOY?"

BY MARCUS L. WARD, D. D. SC.

Read before the Michigan First District Dental Society, May 14, 1908.

Had I known the extent of the cast gold inlay contagion when your committee approached me regarding this subject, I presume it would have been with far greater hesitancy that I consented to present it. The task, however, seems one of duty, and a duty I am not at all reluctant to perform. There was a time when the merits and demerits of amalgam as a filling material caused a great deal of bitterness, but it is doubtful whether the strife and ill feeling engendered between the contending parties of that time ran so high as to bring about a more complete professional ostracism than which is now the fate of those who dare to advocate its use in the presence of the cast gold inlay enthusiasts.

The new opponents still look upon amalgam as a material destined to bring degradation to those who longer use it, and even set at naught the achievements which have been wrought with other filling materials. We often hear gold enthusiasts make the assertion that the men who use large quantities of amalgam are hardly worthy of the name of dentist, while investigation reveals that many who are being thus characterized have simply been less fortunate in securing a wealthy clientele, and are really among the most worthy practitioners.

It is true, however, that many times a mouthful of alloy work represents pure laziness or inability to do better work, though this statement must not be applied when forming an opinion on all cases that come under our observation. Not long ago I heard one of the best qualified men in this vicinity say that he did not feel half as grateful toward the instigators of the porcelain fad as he should if he had been told where NOT to place it, and that it took him some time and caused him some chagrin to learn that it could not be placed successfully in all teeth. In the near future the same opinions may prevail regarding the gold inlay. The contempt now heaped upon the less fortunate or more conservative who can not see their way clear to insert more gold inlays will, it is hoped, turn into greater skill and ability to detect the undesirable qualities of new materials without having to suffer the humiliation resulting from experience. Unquestionably the recent developments in gold inlays will lessen the amount of alloy used, and possibly practically exclude it from a few practices, but amalgam is certain to remain one of our useful and valuable filling materials.

One cannot understand why some operators permit themselves to go into ecstacies over some new operation simply because enamored of its novelty or of some casual use for it. Why not give every subject more profound study, or ask ourselves the question, "What are its undesirable qualities and what is it good for?" We know that the ideal is never reached; that almost every known thing has undesirable qualities, and that sooner or later they will be found out. Then why allow the mind to become absorbed in exaltation, and the actions controlled only by some new or exciting operation or material? Why concede that to introduce or discuss a new thing we must go temporarily insane over it? Why present the subject of porcelain without giving the undesirable as well as the desirable properties? Why advocate the use of drugs for specific cases without giving their dangerous properties? Why advocate removable bridges without describing their limitations? Why laud any particular kind of dentistry, operative, therapeutic or prosthetic, without a word of warning as to what it is good for, and what it is not good for?

When the designer of some new preparation presents to the physician his product, he immediately confronts the questions, "What is in it," and "What class of cases is it good for?" Any attempt on his part to present a "cure-all" would put his preparation into disrepute. Imagine, if you can, some one attempting to introduce a single tonic that met all the demands made upon such a drug. Imagine a similar condition regarding cathartics, when we all know that each of the many that we might name has its particular organ upon which it selects and operates most actively. Imagine, if you will, the schools of engineering pursuing a course of periodically and almost spasmodically introducing materials which had not been tested carefully to select the particular use to which they may be put, and yet we frequently find ourselves in the midst of just such a state of affairs.

Even with the introduction of more scientific methods of manufacture, preparation and manipulation of almost every known thing to-day, the engineer, the physician and the dentist have not found that material or process which meets the requirements of every case to the extent of excluding everything else. Even with the more exact knowledge which we have now of the nature of the physical, chemical and therapeutic properties of the materials we are using, we are only able to apply them to particular pathologic conditions, and then with only a reasonable assurance that they will fulfill the requirements of the particular state or condition for which they have been applied. The man to-day who sets up any one thing as a unit of reference, who assumes to advocate any form, type, example, instance or even combination of conditions to meet the requirements of every case in his practice, or of every man's practice, is certainly in search of an early professional grave. The world is not developing in that direction.

I know of no time or place more appropriate than this, to enter a plea for the development of a keener sense of observation and execution of more conservatism in dental practices. It is not my purpose to enter a plea for anything that will retard our progress as a profession, because we always find enough men whose very nature it is, to oppose innovation and change of whatever nature it may appear. We also find a multitude, who, from prejudice or lack of foresight, are always impeding progress. It is the ultraists of the other extreme to whom we must direct our plea for conservatism. It is the class of men who show no disposition whatever to maintain and adhere to the established order of things, until by study, and observation upon carefully selected cases, the objectionable features which every known material of process possesses, become manifest.

Similar comment may be made upon the sense of observation possessed by dentists. We find too large a number who have not even a remote idea of how to subject a material or process to systematic inspection and scrutiny, for the purpose of ascertaining its laws for practical, let alone scientific purposes. On the other hand, we find many who seem unable to distinguish between the dental office and a laboratory for investigation. They are not satisfied unless something new is being tried out in the mouth which should have been tried out elsewhere. If placed in the right channel, they would make better investigators than dentists. They can see some things which they have been trained to look for, but they fail to observe others of even more importance, for the reason that they do not know the kind of things to look for. The logic of events bears no other interpretation than that this incredulity regarding the necessity for clinging to recognized standards, and lack of a keen sense of observation, are largely responsible for the little significance and interest given to many of our current contributions.

It is manifest everywhere. Men are continually asking what is the best gold to use. What is the best alloy to use? What is the best cement to use? What is the best porcelain to use, and so on through the list of materials we use, as though there had been, or was quite likely to be, something developed which would meet all requirements of such a material to the exclusion of everything else. Such will never happen. We may as well begin now to cultivate observation and conservatism, that we may give our patients the best possible service, and fit ourselves to detect some of the things which at one time signify nothing, but at others become obviously threatening. In order to measure the merits and demerits of any filling material, it is necessary to have recourse to some standard established by custom, public opinion or scientific investigation, by forming a comparison with which we are able to determine whether a material is good or bad.

This standard we find in the form of a number of requirements some of which are of much importance, while others are of only secondary importance. First, a material which is desirable as a filling material must be indestructible in the fluids of the mouth. It must be free from shrinkage and expansion after being made into fillings. It must resist the forces of attrition and mastication. It should be adhesive. It should be a poor conductor of thermal impressions, and probably least important of all, it should admit of easy manipulation. Many other requirements might be added to our list which we shall use as a standard, though they would undoubtedly be as varied as are individuals, and serve only to complicate our task of convincing you that there is no ideal filling material. A similar standard is necessary to enable the physician to administer drugs intelligently, or the engineer to perform some of their modern feats, which are wonderful to say the least.

To the uninformed in this respect, if we were to ask the question, "What is the ideal styptic?" the answer "Adrenalin chloride" would undoubtedly result, while were the same question asked of those informed on the subject, the answer would be adrenalin chloride for temporary contraction of the blood vessels, and tannic acid, alum or salts of iron, for a permanent contraction, for the reason that the former simply excites a contraction of the muscle fibre of the circulatory system, which is only temporary, while the latter coagulates the blood and albumin in the tissues to form a much more permanent contraction. Adrenalin chloride contracts the blood vessels, but they afterwards dilate and remain so. Is it an ideal styptic? No, though it serves us well in many cases. Does it meet all the requirements which form the standard for measuring such drugs? No, not any more than any other drug does. We might carry this kind of discussion all through the drugs that the physician is compelled to use, and the result would be that not one drug could be called "ideal." They would all have their undesirable qualities, and yet we would make much use of them through their desirable ones. Suppose we were to ask similar questions of the engineer, regarding the materials he uses to erect his great structures or to carry on his great commercial enterprises. Would not the absurdity of such questions become the more obvious? Would we not be more firmly convinced that no one thing met all requirements, and that every little change in conditions necessitated a change in materials in order to get the best results?

Gold has for years held first place as a filling material, and justly proud have the men felt who could exhibit those beautiful gold fillings which have given years of service. But why has gold been placed first as a filling material? Does it meet all the requirements of the standard which we have set up to measure filling materials by? Far from it. It is indestructible in the fluids of the mouth, it resists the forces of mastication, it is free from shrinkage and expansion and is sufficiently easily manipulated, but it is a good conductor, a very conspicuous material, and it never adds to the strength of the tooth through the methods by which it is inserted. It has been placed first as a filling material principally because it saved more teeth than ony other material, and it saved more teeth because it is free from shrinkage and expansion and resists the action of every re-agent found in the mouth. Secondarily, it has been placed first because of its intrinsic value and because it, in the past, has represented the highest type of technical skill. You will observe that the material which has so long stood foremost has *several undesirable* qualities, that it is not "ideal" and that it is being crowded out of the anterior teeth because of its appearance, and out of the larger cavities in the posterior teeth because of the lack of adhesiveness, which is now furnished by the gold inlay filling. These two undesirable qualities which we have overlooked in the years past, are now the very ones responsible for the lessened usefulness of gold foil as a filling material. If we carry the discussion on to measure the different golds with a view of determining whether any one of them is ideal, we meet with the same result. Some beautiful results are obtained by the advocates of the numbers 30, 60 and 120 foils, and each one is as certain as can be that his results are best. Is it because the heavy foils are best? Most of us would agree that such foils are best to place on the wearing surface of fillings, but we would insist that much had been sacrificed in the way of adaptation if a whole filling had been made from such foil in a remote part of the mouth. Just as certain are the advocates of the sponge golds that they can perform feats that the heavy foil workers would not even attempt. Most of us would agree that the maximum adhesion and adaptation had been obtained in some of the sponge golds, but would not attempt a comparison of the wearing qualities, for we know that much has been sacrificed in this respect. Does not the significance of the questions "What is the best gold?" and "What is the best filling material," become quite apparent? Is it not clear to us that the word "best" means "most advantageous," rather than "perfect?"

Porcelain may similarly be measured and found to meet all our requirements, except resistance to the forces of mastication. This, however, is of such importance that it becomes necessary to exclude it from all occlusal and most proximo-occlusal surfaces, making it far from that ideal filling material which, from the nature and number of questions asked, so many are apparently looking for. Despite the great claims made by the advocates of some of our high fusing porcelains, that such materials will save as many teeth as any other one kind of filling material, we find upon experiment, confirmed by clinical experience, that such claims are based upon enthusiasm rather than facts, and that the number of adherents to such belief is rapidly waning.

Tin foil has never met with great favor as a filling material, because it does not resist sufficiently the forces of mastication, does not maintain its color, nor is entirely indestructible in the fluids of the mouth. The oxyphosphate cements have never resisted sufficiently the oral fluids nor the forces of mastication to command a prominent place in the field of filling materials for permanent operations.

Amalgam, as furnished us to-day is unquestionably a better filling material than it has been at any time in the past, though the material still stands in a class by itself by being on the border line between what we term materials for permanent operations and those that we term materials for temporary operations. It represents a material which, when compared with some materials may be regarded as permanent, but when compared with others, must not only be regarded as temporary, but inferior in esthetic and hygienic qualities during the time that it preserves the tooth from further caries. Not one of us has failed to observe that some change in our alloy fillings has taken place a year or two after being inserted. Almost every one has inserted fillings which were quite beautiful when polished, many of which have approximated more closely the natural color of the tooth than the gold fillings, which have for years past represented the highest type of repair to damage done by caries. Some of them have remained bright in nearly all mouths, while others of them have turned dark in nearly all mouths. Some of these fillings have remained in the teeth and preserved them from further caries for many years, whilst others have been a disappointment in this respect.

Many of the alloys designed previous to 1895-6 have furnished a great deal of galvanic disturbance, while most of those designed since that time have been practically free from this very undesirable quality. Obviously, we have had furnished us alloys which would save teeth at the expense of color and freedom from galvanic action. We have, likewise, had alloys furnished us which remained bright and been almost free from galvanic action at the expense of saving teeth. If we are to answer the question "Which is the best alloy?" we must first ask the question, "Which quality do you regard as being of supreme importance?" Do you want to save as many teeth as possible with an alloy which in most mouths will turn dark, and in some mouths turn jet black, as well as give galvanic disturbance in a great majority of cases, or do you want to use an alloy which remains bright in almost all mouths, and which is almost free from galvanic disturbances, except during the earlier stages of setting, though it will not save as many teeth? Do you want to learn to work some of the hard-working, rapid-setting alloys with their desirable qualities, which are dependent upon their rapid setting and hard working, or do you prefer to use an alloy with fewer desirable qualities, but works easily and sets slowly? Until we understand that the alloy which saves the most teeth is not, nor cannot be made at present, the best color, that the alloy which gives the most galvanic disturbance is usually the poorer color, though usually saves the most teeth, and that the alloy which sets slowly and works easily is the weakest and least stable thing of its kind we can use, the real meaning of the word "best" does not manifest itself.

I think we shall all agree that the operator who does many gold fillings will best serve his patients if he keeps at hand not one form of gold, but at least three kinds, viz., one of the sponge golds, one of the gold foils about number 4 and one of the heavier golds, either number 30 or 60. There is no doubt but that a skillful and judicious distribution of the three kinds will give far better results than the adherence to any one form, regardless of the talent the operator may possess. The same thing is true with alloys. The author has heard the word "best" in connection with filling materials until the word has become positively distasteful to him. It is an appropriate term to use when speaking of an engine, cabinet, lathe or anything else that the dentist uses of which he cannot well have two in his office. But in no sense should he feel that his best services are being given to his patients by clinging to one gold, one alloy, one porcelain, one cement or anything else that is as easily kept as these things are.

If we are obliged for some reason or another to confine ourselves to one alloy, one gold, etc., let us understand thoroughly that the best we can select means the one of greatest advantage, usefulness or suitability for the purpose intended. That it is the one, *all things considered*, which is best for the man who uses one kind only, and must not be considered the best material for all cases. It may be far from it.

We have had, since the work of Black in 1895-6, two great classes of alloys furnished us, one of which represents more closely his ideas than the other. Quickly did the manufacturers take advantage of one of Black's discoveries, viz., that 50 parts silver and 50 parts tin, first shrank 2 points and afterwards expanded 2 points, and placed upon the market the class of alloys represented by Par Excellence, Ductile, Success, White Alloy, Standard White, Crescent, Sibley's G. & P., Revelation, etc., principally to catch the trade that wanted an alloy which set slowly, worked easily and remained a good color. If we measure these alloys by the standard that we have set, we find that the good qualities have all been mentioned except that this class of alloys are almost free from galvanic disturbances.

The most undesirable qualities of this class of alloys are lack of strength and failure to lie still after being inserted as fillings. About 2 years ago the author brought strongly to the attention of the profession, the fact that many of our alloys would continue the movement after being inserted, which accompanied their earlier stages of setting. At that time it was necessary to condemn them and question seriously the excuse for their being. Since then more evidence has been added to confirm the theories then advanced. The real foundation for the unstability of this class of alloys more than other classes, is the fact that they unite with mercury easily. They require little working to make them plastic enough to be inserted. Obviously, if they require only a little working outside the mouth, and a certain movement accompanies this working, they will be just the ones to move easiest after being inserted.

In other words, the alloy which is the most stable in the mouth, is the one which works hardest outside the mouth. We cannot expect an alloy that is so soft that it will unite with mercury with little or no trituration, to be as hard and stable as one which requires vigorous working. These alloys will save teeth, but their life is shorter than those which work harder. If the question is asked, "Which is the best alloy?" will you accept the answer, "Something that works harder," or will you place your individual likings regarding easy working alloys against more permanent results? In other words, "What qualities are of most importance to you?" You must determine which quality you regard as paramount, and learn that stable alloys are not easiest worked. With their extremely high percentage of tin, which, as you all know, is a very poor conductor, this class of alloys scarcely ever gives any discomfort commonly attributed to galvanic irritation. The total absence of copper also adds to this quality, since it is not only a good conductor, but is readily acted upon by the oral fluids. Much more might be said about galvanic disturbances, a phenomenon which once accompanied the use of nearly all alloys, but to-day is almost unheard of. Suffice it to say that if this quality which this class of alloys possess above all others,is of supreme importance to you, or if you place more stress upon an easy-working alloy than you do upon permanency of form, choose one of these alloys. The leading manufacturers' products are practically all alike. There is, in reality, no best among them.

The other class of alloys which have been furnished us since 95-96 are known as Black's Alloys—Rapid-setting alloys and high silver alloys. The three names by which this class is designated are used synonomously. They contain about 20 percent more silver, 23 percent less tin and about the same amount of zinc as the soft alloys. In addition to the same constituents, they contain about 5 percent of copper, a material which always has been, and promises to be indespensible in our most stable alloys.

Like the plastic alloys just mentioned, they are not only the product of the students of Black, but of almost every leading manufacturer who has a chemist and metallurgist capable of imitating his work. They are represented by such alloys as the 20th Century, Rego, Fellowship, Micrometric, Acme, True Dent Alloy, Permaneo, Triumph, Superior, etc. They are, as you have all observed, practically unacted upon by the oral fluids which results in being quite free from galvanic irritation, and allows them to maintain a good color. They resist the forces of attrition and mastication quite well, though are not to be compared with gold, and, in some respects, porcelain. They are, unfortunately, good conductors. They are as free from shrinkage and expansion as it is possible to make them with our present knowledge of this material, and give a product which is strong and maintains a good color, though this movement, little as it may appear, is sufficient to make them much poorer tooth preservers than other materials which lie absolutely still. They are not entirely free from shrinkage and expansion, and cannot be made so without inserting larger quantities of copper, which would not only destroy their color, but afford a greater opportunity for galvanic irritation, thus bringing us back in some respects to what we had a few years ago, viz., alloys which might save more teeth, but were of an inferior color and subject to galvanic irritation, though we would have the advantage of being able to produce a fairly uniform product. You ask why not use something else than copper, and the reply is, they have all been tried and nothing been found. If the question is again asked, which is the best alloy, the only reply is, Do you want to save teeth at the expense of other things which are just as essential. These alloys are not quite as light colored as the plastic alloys, and cannot be made as light without the removal of their copper and some of their silver which would at once destroy their greater hardness and permanency of form. The farther we delve into the question the more plainly do we see that these alloys which are unquestionably the best, all things considered, are not perfect. They are not the best tooth preservers, they are not quite the best color, nor are they the most free from galvanic irritation.

Of course an entirely different view of the question "Which is the best alloy?" may be taken, though it is one which must be considered with care, and our decision rendered, not in the midst of enthusiasm, but after the opinion of each of our most enterprising manufacturers has been carefully considered. There are numerous makers of alloys whose opinions we feel are not worthy of consideration, though they are not among our leading manufacturers. Our leading manufacturers to-day are making each of the classes of alloys mentioned as nearly as perfect as their chemists can do it. But to make the question more pertinent, "Is not one chemist more capable than another?" Undoubtedly this is true to some extent, but it is not as important to us as the variety of opinions held by the various makers of alloys as to what really constitutes the best alloy. It is the same *old* question "When we cannot meet all the requirements, which ones shall be given the preference?" Shall it be color, shall it be hardness, or shall it be to save teeth?

The very first operation necessary in the production of an alloy, viz, the melting of the constituents, is one which not only the dental chemists, but many others differ markedly upon. The rule usually followed in such procedures is to melt the constituent first which has the highest melting point and follow with the others in the order of their melting point, reserving the most volatile ones to the last. If this rule is followed out by melting the copper at 1050 degrees C., then the silver at 960 degrees C., then the zinc at 419 degrees C., and finally the tin at 238 degrees C., the result is, even in small quantities with a perfect reducing agent, a loss in weight. Whether the loss has been caused by oxidation of the copper or volatilization of the zinc, remains for the analyst to determine, and herein lies one of the secrets of being able to furnish you with an absolutely uniform product. No one but the chemist can do it.

Unquestionably the future will prove that this order must be changed to bring out the best results, but until it is decided definitely, and until the temperature at which these alloys should be poured to prevent segregation of some of the constituents, and a strong tendency of the others to crystallize out in excess, will there be dissimilarity in the behavior of alloys towards mercury which are otherwise identically the same.

Your attention is now directed towards another evidence of different opinions of makers of alloys. For example, let us examine the leading alloy made by L. D. Caulk & Co. (20th Century), and the leading alloy made by S. S. White Co. (True Dentalloy). These two manufacturers are selected not with a view of either lauding or criticizing their products, but because they are well and favorably known to almost every one. We find the 20th Century alloy in the market in 2 grades as regards the setting. The package containing rapid setting alloy appears in every way identical with the one containing slower setting alloy. Chemically it is the same, but physically we find a little difference. The one marked rapid setting has been only partly annealed, while the one marked slower setting has been completely annealed. The only difference between the two is that one sets a little faster than the other, and as a result of its setting a little faster, requires a little more mercury. We find S. S. White's product (True Dentalloy) in the market in one grade as regards setting, instead of two, as we did Caulk's product. It is completely annealed, and compares favorably with

Caulk's product marked slower setting. Now, why does Caulk's give you one alloy which sets more rapidly than the other? And why does not S. S. White Co's. do the same thing? Evidently, White's believe that a man should learn to work one grade of alloy instead of two, and should modify the setting by the amount of mercury left in the filling and the amount of working, while Caulk believes that an alloy better be made rapid setting by the incomplete annealing than by the working and mercury method. Both of these companies understand thoroughly that the alloy should be completely annealed, but they differ as to how to meet the demands of the never satisfied dentist, who wants an alloy that sets more rapidly, even if it is known to be a little less desirable in other respects. To decide which of these companies is right and which wrong, would involve such a number of conditions that time and labor only can tell, though I firmly believe that they are both giving us the best that present knowledge of the question will permit. Another evidence of how some of the makers of alloys differ, may be found by observing the difference in the shape and fineness of the communition of some alloys. Some are cut in coarse shavings, some in fine shavings, some in coarse filings and some in fine filings. Some of the makers of alloys have apparently permitted themselves to become as much enthused over the particular character of the cut of their alloys as the dentists themselves have. Unquestionably the shape and size of the small particles of alloy modify the behavior of the mercury upon it somewhat, especially in the earlier stages of their union, but, there is no reason whatever for the dentist selecting an alloy because it is cut in any particular form, except to gratify his individual fancy on how it should feel when it is worked in the hand. A multitude of things might be said in this connection not only to show the idiosyncrasies of the makers of alloys, but the emperical methods practiced by the dentists themselves. Some of these I will now discuss orally, to avoid bringing into print without their consent, the names of certain concerns and individuals.

In conclusion, let me emphasize the necessity for keener observation, if we are to select from the products just spoken of, the ones best suited to the cases that come under our observation.

Let me warn the gold inlay enthusiasts to cling to their time tried fillings till they have carefully tested the gold inlay, and found out whether it is a success in their hands. Let me again remind you that while too much must not be expected from any alloys, we can apply the rapid setting alloy in its place, the slower setting (not plastic) alloy in its place, and occasionally the copper alloy in its place, and obtain far better results from almost any viewpoint than to adhere closely to any one particular kind.

Let us all be vigilantly attentive to the new things as they develop, but at all times keep our enthusiasm moderated with good judgment.

### DISCUSSION.

Dr. Buttrick: Dr. Ward was kind enough to give me a copy of his paper yesterday. So I took it home and read it over in the evening and after "digesting" it I have made a few notes which I shall give to you, although I do not believe that I can add anything of value to what has already been said.

The essayist was a good one to choose for this subject as he has given a good deal of time to his researches along this line and I am sure that what he has told us will aid us in making a choice of an alloy to use in our several practices. I presume however, that our successors will still be discussing this question long after the grass is green above us.

I think he should have taken it for granted however that each one of us wished to secure the alloy that would be best for his patients without regard to any other consideration and I am convinced from his statements both here and upon former occasions that the so-called high-silver or rapid setting alloys made after the Black formula do serve the patients best all things considered. In his paper the essayist has said somewhere that several forms of filling material—say of gold for instance were best for those who were still inserting gold fillings. I do not believe however that he would suggest several different alloys. My opinion is that it is best to select one alloy and then stick to it, thereby learning its working qualities and also its limitations. The question of price should never enter into this nor any other thing of this kind that you must buy.

I am not going to take up the cudgel in defense of that long suffering and much despised amalgam, but I am not ashamed to say that I use it in my practice and think that it has its place in every well regulated dentist office. Those who protest glibly in public against its use are still employing it in their own practice and doubtless will until the end of the chapter. I was somewhat amused last summer when a patient of one of those who so despise amalgam came into my office in the absence from the city of her own dentist with a tooth that was troubling. This dentist had just finished doing some work for her and had pronounced her all safe and sound again. Absolutely all the work he had done was in amalgam although in my estimation some other material would have served the purpose better and there was a larger approximal cavity in one of the lower bicuspids which had escaped attention altogether.

I think the essayist has well spoken for more conservatism in taking up with new fads. Try each thing out well in carefully selected cases and then adopt or reject it. Those who a few years ago were advocating the use of porcelain everywhere regardless of its deficiencies will be found in the van guard of those who can now see no places where gold inlays are not indicated and next year some new fad will engage their enthusiasm. I think the dentist that best serves his practice is he who can from a full knowledge of all possible filling materials select that one best suited to the case in hand under the conditions as they exist.

We are none of us in the practice of dentistry from philanthropic motives, and the one question which is ever a very present one with most of us is: "What can this patient afford?" If a particular cavity might be filled with either a gold inlay, a gold filling or an amalgam, that one best serves the patient that comes within his means. A dentist would be a fool to put in a gold inlay at the price of an amalgam filling, and he would be equally foolish to put in a gold inlay when the patient could not really afford it. There are some dentists who degrade the profession because they cannot see beyond the almighty dollar. They will recommend that which will pay them the most money regardless of the best interests of the patient who has trustingly put himself in his hands.

We know that the adhesion of the cemented filling gives added strength to the tooth and that the color of amalgam is against it but I think one of the worst features of amalgam is that it can be so easily worked. The result is the sloppiest kind of work in many instances. This has done much to bring discredit upon this much abused material. Look well to your cavity preparation and see to it that your contours and contacts and occlusions are perfect and you will find amalgam a tooth saver of great merit. If a thing is worth doing at all it is worth doing well.

When the question of price does not enter into the question at all, learn to select that material that will best serve the patient at hand. This reminds me of the physician who ordered his patient "to take something or other with brains." In last evenings paper the president was said to have gotten himself disliked in some high quarters by saying that some senator had "sweetbreads for brains" and I fear from some of the results that we see in the mouths of some patients that some dentists might come under the same arraignment! Not only must price and toothsaving quality be taken into consideration but the condition of the patient as well. In my own mouth I know that I do not want extensive and painful cutting to accomplish an ideal. "Extension for prevention" is well enough perhaps when the other fellow is concerned but try it in your own mouth sometimes and see the difference. Furthermore with prophylaxis it is not necessary.

Amalgam is a boon in the cases of children and some nervous patients. It always seems to me wrong in the extreme to exhaust a patient for the sake of an ideal operation.

Furthermore so-called permanent work cannot be done in the mouths of children and others where decay is rampant and expensive operations are often contra-indicated, while on the other hand almost any old thing will suffice where the tendency to decay seems abolished or a certain immunity is established. While prophylaxis is of undoubted value, I do not think uncleanliness is the chief factor in dental caries. While we should always strive to do ideal work and amalgam work will never be ideal, still in my estimation it occupies a very useful field and one which will not be disputed by any of the present candidates for favor.

Dr. C. H. Land: It is not often that a man is allowed to see amalgam filed from copper, silver and tin, and has watched its progress from that time on, to the present time to see what the results were. It is not often that he will live to see the results, but it so happens that when I started in dentistry the only way I could get amalgam was to file and make it myself—by filing the silver, and tin, taking a 25 cent piece and cutting it up in that way. Those old fillings turned black, but they saved a great many teeth. The modern amalgam certainly does not shrink as much, and its color is better. Its strength is better. That much you have gained, but there is one thing you always seem to lose sight of. I tried many experiments, made all forms of amalgam, and have put some amalgams on the market to sell them, but to show you the way that I studied, I have here in this little glass tube alcohol that has been corked in for fourteen years and it has not leaked out yet, so there are such things as non-fusible amalgams. I have here specimens shown at the Connecticut Valley Dental Assn., amalgam adhering for fourteen years to plate glass. I have here also another specimen that has been fixed in a different way. It is a wine-glass adhering to plate glass. I wanted to show you these to let you know how I studied the matter. What does amalgam lack more than anything else? It lacks adhesion to the bone. It has practically no adhesion, it may have say, ten per cent. Here is a manifestation of adhesion to glass, and the singular part of these amalgams is that they adhere to the teeth so close that they injure the teeth, why, I do not know. Those that have tetanium in them, or too much bismuth make the teeth decay. I began to realize there was something wrong with the chemical action of the amalgam—what that was I had not time to analize. I came to the conclusion that if I retained that adhesion to the walls of the teeth with a preserving medium I would save the teeth. I have mouths where I have filled teeth with amalgam that has been in 33 years, and no shrinkage whatever. They are held in by cement. For my amalgam fillings that I put in at the present time I mix 10 to 20 per cent red sulphide of mercury in my cement, and then burnish the amalgam to the walls of the teeth with that, and it will not leak. It is this in the tube that has prevented the alcohol from leaking, and it is the only kind that I have found that would hold. All the other amalgams will let the alcohol out in less than six months. That is simply an alloy of gutta percha—that stops the shrinkage. There is evidence of it—14 years in that tube—so that the amalgam that I put on the market was an alloy, gutta percha and amalgam, made very similar to those made to-day.

The proper view, to have of amalgam is practically durability. There is nothing aesthetic about it; there is nothing adhesive about it unless you create that adhesion artificially. The proper way to make it adhesive is to use an amalgam with ten or fifteen per cent of chloro-percha for the interior; burnish that to the walls of the cavity. That will hold without any cementing. If you like it to stay longer and not shrink, then finish with your hard, quick setting allow on the outside for high-finish. If you will do this, all these troubles of shrinkage, of these troubles of not being strong enough to resist the forces of mastication will only be handicapped by how you select your case, and if you want to prevent that, there are ways to do that. I have had some wire mesh made from tin, very thin, and some made from brass wire, similar to bell metal—as fine as made for filtering milk,—which I cut into small pieces and then mix that in with the amalgam. We should select amalgam for practical work, not for the aesthetic, it is one of the most useful fillings of modern times, but you must always draw the line between that which is aesthetic and that which is practical. For the poor, for saving of time, saving of pain and saving of annoyance, it is more valuable to use, and a man has more right to use it than to put his patient to the frightful pain of those long operations of any description. When it comes to the aesthetics you have one filling which reaches nearly to the ideal, that is porcelain. When anybody says that porcelain will not stand the resistance of mastication, then that individual does not know how to make it. Gold is weak compared with it. It is a question of how thick you can make it, and where you are going to put it, when you can reason this out, and use the proper material, gold is not any comparison with it for permanency or durability, nor for the aesthetic results.

Dr. Bowles. Dr. Ward said that the individual must go into this matter of filling materials, therapeutic remedies, etc., and study it for himself. I suspect from Dr. Ward's standpoint that is correct, and yet it is not the individual study of the mass of the profession along these lines from statistics that brings about any particular progress. We study our own cases and draw conclusions, but they are not based on correct data. Very few of us have scientific minds, and very few of us make notes, and we do not know. Dr. Land says, if you will do as he says, use certain preparations, that it will prevent caries more than some other remedy. How are you going to know? The average man does not have any statistics that will tell him that one alloy will save teeth more than another alloy. I think the average man is not prepared, from the investigations he makes, to make any definite statement of that kind. Conditions change. In some mouths an old filling filed up from a quarter may save the teeth for 20 years perhaps, and at the end of 20 years perhaps the best alloy that was ever manufactured might be put in that mouth and in a year or two it may be all gone. It may not be because of the preparation used, but changed conditions in the mouth. So many things of that character have to be taken into consideration that observation, it seems to me, is not very reliable. It is only on scientific investigation in the laboratory that we get anything of definite character. It has been well said that the best material for any man to use is that with which he can do the best work, and in that regard of course we have got to make individual study. We have got to do the operation with the material and in the way that we can do it the best. Some men put in gold fillings almost anywhere and in any old kind of a tooth, where it has to be braced in order to make the tooth solid enough to hammer in the gold. I cannot do that. I can save that tooth better with cement or amalgam. The individual skill of course, has a great deal to do with matters of this kind.

One question I want to ask Dr. Ward, regarding the qualities of the quick setting alloys. In a paper he read in New York some time ago he stated, as he has tonight, that the quick setting alloys are not as greatly, or thoroughly or completely annealed as the slow setting alloys; and the setting of any alloy when it is first put upon the market as a quick setting alloy will in perhaps a year become a slow setting alloy, and after they are kept perhaps two years they are worthless. He has stated that the quick setting alloy requires more mercury, and that the more mercury in the filling the poorer the fillings, because there is more change; that until that mercury has become amalgamated completely by the molecules in the alloy there is going to be change. I would like the doctor to enlarge on that point.

Dr. C. P. Wood: In large fillings or roots, where you want to build all the way round with amalgam, what is the effect of building on one-half with one mix of alloy, and then making another mix to finish the structure? Is it a practicable thing to do? I have been obliged to do it in some cases. As I say, I built the whole crown up with amalgam for abutments, and I have not been able to find any disadvantage in that, and yet I did not know but there might be some bad effect on the one part or the other of the filling in the two mixes. I use rapid setting alloy and to build a whole crown in that way for abutment frequently I get caught where I cannot finish and have to make another mix and put on more.

Dr. Ward. I would like to say in reply to Dr. Bowles' suggestion as to the inability of the dentist to observe things as he goes along. Now I cannot help but feel that we are so far behind the physicians in that respect that there is not really much comparison. The alert physician of today will ask you, the first thing you mention to him: "What is in this thing" or what is in that thing, and what is its action. There is not a physician today who is watching developments in new things who ever had a good training that will do anything of any description with an unknown preparation. He wants to know what's in it and what its action is, the first thing. I have every reason to believe that there are three alloys which have been practically excluded in this state in three years through a process of education. The men have been asked repeatedly to look after two or three things, expansion and shrinkage with which the alloys work, and they have decided to do so. The third one, the manufacturers' agent complained bitterly of their having no sale for plastic alloys, and I think it has been brought about through education. I know the last three or four classes of students that have gone out from Ann Arbor, have seen experiments made in the laboratory, and I am sure none of them will use one of these plastic alloys because of what they saw. I believe that the dentist is able to detect the difference between what is commonly known as the black ditch and what is expansion and shrinkage; I beieve he is able to detect, the first time he sets his eyes on the alloy, whether it is setting too rapidly or not. I believe he can state inside of two weeks whether the alloy has diminished in the mouth and what is the result of too much diminishing. It will save more teeth than anything else you can get, but it had two metals, silver and copper, the best known conductors in there that was a source of all the inaction that came from those old troubles called "galvanic radiation". I believe a dentist can detect these things as well as a physician can detect the action of drugs, and I believe dentists in ten years from today will not be using any more of the plastic alloys. They will be very few, if any at all. I believe men can see in a year or two that the plastic alloys have changed all out of proportion, and oftentimes they almost double their bulk. I believe that the education of dentists is the first thing to do. I do not believe that you can go out and say to a dentist, use this or that. I think he must learn to know why it is better, and the other poorer, and until they have done this there certainly will be no uniformity in a selection of material surely.

In regard to Dr. Bowles' suggestion as to the amount of change which takes place in the rapid setting alloys and those requiring more mercury, I will say that that is the very thing which is being dwelt upon at the present time by three or four of us, pretty strongly; that is, whether or not the quantity of mercury that is left in the filling has a great deal to do with the amount of expansion. We know that a certain quantity is sufficient to put the whole into a solution, that any certain quantity will be taken up; we also know that after this mercury has been taken into certain fillings that it will leave those fillings by two processes: First by reduction, such as pounding or pressure. Whether the mercury after causing the expansion from one will soon creep up in other parts of the filling, by the affinity of these metals we are unable to tell, but we expect something to develop in that line soon. Now, then, speaking generally, the amount of mercury that is left in the rapid setting alloy has a great deal to do with the amount of expansion that takes place, and we cannot say that it is all out for the reason that the mercury which is necessary in there may cause a further expansion by changing places from one part of the filling to the other. Mercury, as you know, leaves all its alloys by a little heat, and it leaves almost all of them by pressure. Now with the plastic alloys like the Success, Revolution, Dugdale and that class, it only takes a short time; possibly a year will almost complete the union of the silver in that alloy with the mercury for the reason that there is nothing to inhibit this union except a great mass of tin, about fifty per cent. Staining with iodine shows that mercury will travel in one of these alloys in a great deal shorter time than in the slower setting, because of a larger amount of tin, etc.

In reply to Dr. Wood's question, would say that the tests in which one alloy is built upon another show that there is no known process why you can change shrinkage to expansion nor expansion to shrinkage. If it is bound to expand it will expand; if it is bound to shrink, it will shrink. You cannot change it by changing its composition, or by working. We know of nothing at all that will change one movement to another. We can modify the amount by annealing and working, but cannot change one movement to another. The principle difference in results is in strength. If it is fresh, it will adhere to the tooth, but aside from the variation in the strength I know of no difference that you would get by mixing it by any one means. If it is strong, enough, that is the principal thing.

There was one other thing that possibly I did not make as plain as I should, in regard to the choice of other alloys. I cannot use a rapidly setting alloy in all cases of similar kind, but at the same time I want it where I can get it. I do not mean that you want one of those plastic alloys; they are the poorest risks of anything that I know of. I feel, however, that one should have such an alloy as Acme, True Dent, or Sterion White for places difficult of access. I can possibly in desperate cases use to advantage a copper alloy, although it is of black color and subject to all kinds of galvanic disturbances. I do not mean that a man should have four or five alloys and not know how to manipulate any one of them, but I believe he ought to have two slow grades of setting, but the slower setting alloy is the one which is most nearly perfect.

